# Simplifying Knee OA Prognosis: A Deep Learning Approach Using Radiographs and Minimal Clinical Inputs

**DOI:** 10.3390/diagnostics15192543

**Published:** 2025-10-09

**Authors:** Cheng-Tzu Wang, Kai-Ting Chang, Feipei Lai, Jwo-Luen Pao, Shang-Ming Lin, Chih-Hung Chang

**Affiliations:** 1 Department of Orthopedic Surgery, Far Eastern Memorial Hospital, No. 21, Section 2, Nanya South Rd., Banqiao District, New Taipei City 10617, Taiwan; jwoluenpao@gmail.com (J.-L.P.); orthocch@gmail.com (C.-H.C.); 2 Department of Mechanical Engineering, Asia Eastern University of Science and Technology, New Taipei City 220303, Taiwan; fc013@mail.aeust.edu.tw; 3 Department of Computer Science and Information Engineering, National Taiwan University, New Taipei City 10617, Taiwan; kevin1997324@gmail.com; 4 Graduate Institute of Biomedical Electronics and Bioinformatics, National Taiwan University, New Taipei City 10617, Taiwan; feipeilai0517@gmail.com; 5 Graduate School of Biotechnology and Bioengineering, Yuan Ze University, Taoyuan 320315, Taiwan

**Keywords:** Kellgren and Lawrence classification, knee OA prediction, deep learning

## Abstract

**Objectives:** To predict the progression of knee osteoarthritis (OA), a deep convolutional neural network model was developed and applied to basic images and clinical data. **Design:** A vision transformer-based model was trained using 5565 knee radiographs as baseline images from the osteoarthritis initiative (OAI), including 578 testing images. Each knee had a corresponding Kellgren and Lawrence (KL) stage after 48 months of follow-up. Another 274 cases from the Far Eastern Memorial Hospital were used for external validation. The data included a combination of single/pairing images and full/essential clinical factors. Area under the receiver operating characteristics (AUROC), accuracy, sensitivity, specificity, odds ratio, and ability to discriminate surgical candidates were applied to evaluate model performance. **Results:** In cases with OA progression, the AUROC for identifying surgical candidates was 0.844, 0.804, 0.766, and 0.718 in the combination of a single image with essential factors, single image with full factors, pairing images with essential factors, and pairing images with full factors, respectively. In OAI testing using the simplest input, AUROC of identifying OA progression was 0.808, with 74.1% accuracy, 91.8% sensitivity, and 71% specificity. In external validation, AUROC of identifying OA progression was 0.709, with 71.2% accuracy, 72.2% sensitivity, and 70.3% specificity. Positive model prediction had an odds ratio of 23.87 (CI: 11.24~50.67) in OAI and 5.92 (CI: 3.50~10.03) in external validation. **Conclusions:** Our model provides reliable prediction results for knee OA cases with the advantages of simplicity and flexibility. The model performance was excellent in progression cases, potentially making early intervention in OA patients more efficient.

## 1. Introduction

Knee osteoarthritis (OA) is one of the leading diseases inflicting the older population worldwide [1]. First reported in 1957, the Kellgren and Lawrence classification is still the most popular scoring system for knee OA grading [2]. In the early KL stage, the symptoms can be controlled effectively by conservative treatment. However, surgery may be needed for terminal OA because of the irreversible nature of knee OA. Therefore, the identification of cases with a tendency to progress in the future is essential for the effective treatment of the disease. Early intervention including weight loss, lifestyle modification, and quadriceps training may slow OA progression [3].

Many risk factors have been recognized for knee OA, including old age, female sex, higher body mass index (BMI), previous knee surgery, and trauma history [4,5]. Genetic components of knee OA were also identified in recent studies [6,7]. Genetic studies (GWAS/meta-analyses) identify numerous risk loci (e.g., GDF5 and additional non-coding variants), underscoring multifactorial disease biology but also the need for image-based and clinical predictors to capture structural progression [8].

In the last decade, artificial intelligence has been applied for the classification of medical images and diagnosis of diseases by many studies. Both radiographic and magnetic resonance images (MRI) provide useful information for knee OA progression [9,10]. However, the high price and poor accessibility of gene studies and MRI prohibit their widespread application for OA prediction modeling. Recent approaches use diffusion-based generative forecasting of future images coupled with classification, but they typically rely on image-only inputs and introduce substantial computational overhead for image synthesis and interpretability [11]. In parallel, multimodal/tabular machine-learning methods have predicted structural progression by leveraging demographic variables and radiographic grades, with SHAP providing interpretability. However, these approaches rely on clinician-curated radiographic inputs and do not learn end-to-end from raw images [12].

Building on prior works, we present a vision transformer–based model that integrates raw radiographs with clinical risk factors in a single end-to-end framework. The architecture captures global joint context and preserves clinical interpretability via attention maps, complementing image-only generative pipelines and tabular multimodal methods. Our goal was to develop a knee osteoarthritis prediction model trained on a public cohort and externally validated on an independent hospital dataset from Taiwan.

## 2. Methods

### 2.1. Dataset and Participants

The present study used data from the osteoarthritis initiative for model training, validation, and testing. OAI is a multicenter cohort study with 9 years of follow-up. The clinical factors and radiographic images for disease onset and progression are publicly available. The cohort of 4796 participants was aged from 45 to 79 years at the time of recruitment. The cases with incomplete records of clinical risk factors and KL4 stage of baseline X-ray images were excluded from the study. In total, 5565 OAI knee images with accompanying clinical factors were included in our study, as shown in Table 1. In addition, 274 participants from our institute (Far Eastern Memorial Hospital [FEMH], a tertiary medical center in New Taipei City, Taiwan) were involved in the external validation. This study reviewed the cases with knee radiographs that were presented to our institute from 2006 to 2024. The provided classification was based on the initial classification in the medical record (as shown in Table 1). The participants had a baseline knee X-ray image and a 4-year follow-up knee X-ray with 3-month tolerance.

### 2.2. Radiographic Data and Clinical Risk Factors

All radiographic images were in standard standing posteroanterior view. The Kellgren and Lawrence score was applied for knee OA grading in our study. The cases diagnosed as KL grades of 0 and 1 represent no obvious radiographic evidence of knee OA because the clinical significance of these cases is similar in clinical practice. Based on the consensus of the expert committee in this study, KL Grades of 0–1 were classified as “doubtful OA”; whereas, KL Grades of 2, 3, and 4 referred to mild, moderate, and severe OA, respectively.

**Figure 1:**
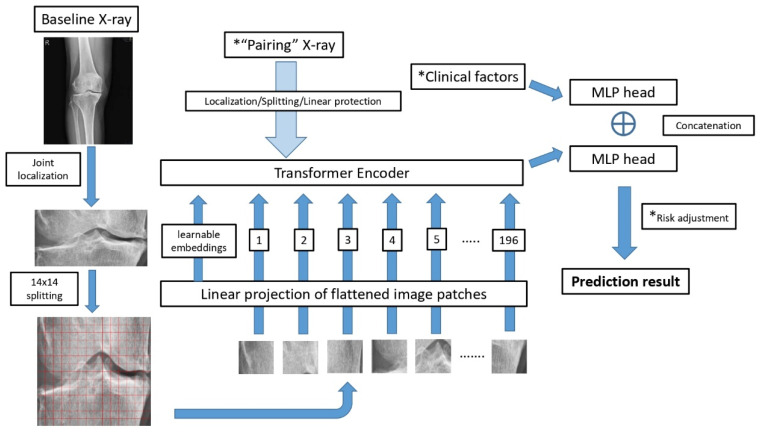
Pipeline and model architecture. * Note there are three adjustable steps in the model design. (1) “Pairing” X-ray is optional if additional images were examined about 1 year after the baseline. (2) Either “full factors” or “essential factors” can be applied as clinical factors before advancing to MLP head. If more information is available, the model can be re-trained easily via this algorithm. (3) BMI was applied for risk adjustment in the present model. This factor can be changed in the future if more available data are identified as significant.

The radiographic data for model training were defined as baseline images. We performed two modes of radiographic input during model training. In the first mode, “single baseline images” of knee X-ray with clinical factors were used to predict the KL stage after 48 months. In the second mode, “pairing baseline images” with an additional knee X-ray were examined 12 months after the baseline image was added as input to predict the future KL stage (Figure 1). The model prediction results were then compared with the 48-month follow-up KL grade obtained from the OAI database.

The clinical factors for model training were based on the known risk factors of knee OA established in previous studies [4,5]. Model performance was evaluated via “full factors” as well as “essential factors” with the full factors including age, gender, BMI, weight, surgical history, knee injection history, medication use for pain, WOMAC knee score, anserine bursa pain or tenderness present on exam, and patellofemoral crepitus. Essential factors included age, gender, and BMI.

The KL grade of baseline and follow-up images and clinical factors were obtained from OAI central reading. The KL grade of external validation was decided by experts in our institute, including two orthopedic surgeons and a radiologist, each with more than 10 years of experience. Baseline risk factors of external validation were collected by medical records in our institute. To avoid overfitting, the baseline KL grade was not input during model training.

The input data for model training included the baseline X-ray image and aforementioned factors consisting of age, gender, and BMI. The model prediction results were then compared with the 48-month follow-up KL grade obtained from the OAI database. (Figure 1).

### 2.3. Model Architecture and Algorithm

A vision transformer (ViT) was used as the backbone of model architecture [13,14]. As shown in Figure 1, knee joint localization was performed to crop a joint-centered region of interest (ROI) prior to modeling. This standardization improved attention localization and downstream performance. Cropped images were resized to 224 × 224 px and normalized per channel (mean 0.5, SD 0.5), consistent with ViT initialization. The vision transformer then ingested each image as 196 non-overlapping patches (14 × 14) with learned positional embeddings. Models were initialized from ImageNet-pretrained ViT weights (pretrained on ImageNet-21k and fine-tuned on ImageNet-1k) and subsequently fine-tuned on our dataset.

The resulting vector sequence was applied to a transformer encoder and subsequently advanced to a multilayer perceptron (MLP). In addition, clinical factors were applied to another MLP. The model then generated an original prediction result after applying concatenation. Several steps were flexible in model tuning and data expansion, including “pairing X-ray images,” “clinical factors,” and “risk adjustment,” as shown in Figure 1. No stochastic geometric or photometric augmentation was applied. Radiographic geometry was intentionally preserved to retain cues for joint-space narrowing and osteophyte formation, and the localization/cropping procedure already standardized the ROI.

### 2.4. Evaluation of Model Performance

The input consisting of image number (single baseline images, pairing baseline images) and clinical factors (full factors, essential factors) was analyzed to select the most effective combination. Accuracy, sensitivity, specificity, AUROC, and attention map were applied to evaluate model performance.

Based on the special importance of recognizing surgical candidates, the power to identify moderate-to-severe knee OA (KL 3 and KL 4) in follow-up cases was assessed by AUROC. We also compared the odds ratio (OR) of model prediction with clinical factors to quantify the prediction power.

### 2.5. Statistical Analysis

Statistical analysis was performed using SPSS (version 29). All analyzed data consisted of statistically independent observations. Statistical significance was defined as a *p*-value of less than 0.05. Discrimination was assessed by the AUROC with 95% confidence intervals (CI) obtained via bootstrap resampling. About between-model comparisons, pairwise differences in AUROC were evaluated using the non-parametric DeLong test (two-sided). We report odds ratios (OR) for positive model predictions using logistic regression, with 95% CI (Wald). For, external validation, no model or threshold was re-tuned on the external dataset; all analyses used the threshold fixed on the OAI validation set.

### 2.6. Ethical Approval

Ethical approval was obtained from our Institutional Review Board (Ethics Committee of the Far Eastern Memorial Hospital: no. 110235-E); it waived the requirement for obtaining patient informed consent due to the retrospective nature of the study. There is no external funding for this research. There is no conflict of interests to declare.

## 3. Results

Table 2 shows the comparison between the OAI testing set and external validation. The OAI testing set had a mean age of 60.6 years with mean BMI of 28.6 kg/m^2^; 237 were male; and 86 knees progressed to advanced OA stage after 48 months of follow-up. The external validation had a mean age of 71.4 years, mean BMI of 25.9 kg/m^2^, and 57 were male. Progression of knee OA during follow-up was found in 119 knees.

**Figure 2:**
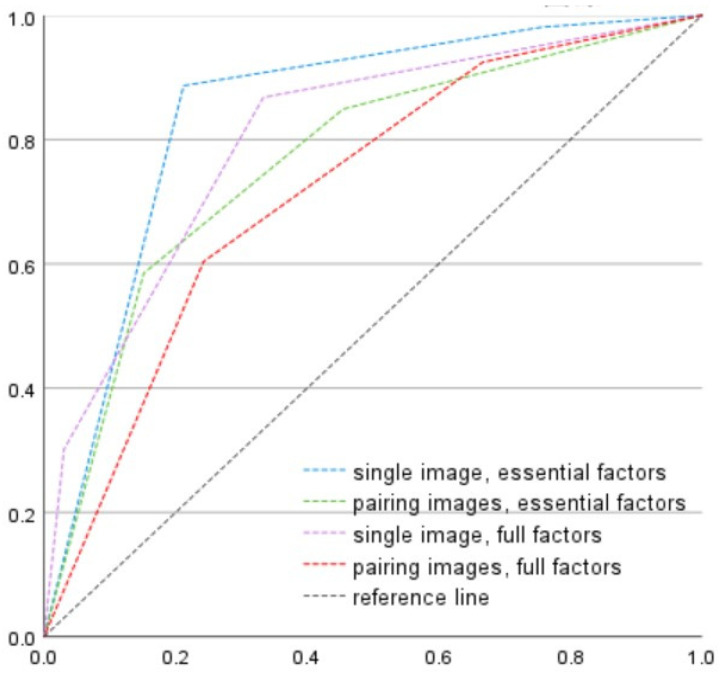
Discrimination of surgical candidates in progression cases. The evaluated cases included (1) Doubtful-to-mild OA (KL1 and KL2) in baseline progressed to surgical candidates (KL3 and KL4) during follow-up (2) Moderate OA (KL3) in baseline progressed to severe OA (KL4) during follow-up. The AUROC was 0.844, 0.804, 0.766, and 0.718 in the combination of single image with essential factors, single image with full factors, pairing images with essential factors, and pairing image with full factors, respectively. The reference line indicates level of prediction by chance alone.

In the OAI dataset, the accuracy of “single image with essential factors,” “single image with full factors,” “pairing image with essential factors,” and “pairing image with full factors” was 74.1% (sensitivity: 91.8%, specificity: 71.0%), 85.8% (sensitivity: 25.6%, specificity: 96.2%), 70.6% (sensitivity: 72.4%, specificity: 96.2%), and 60.9% (sensitivity: 69.7%, specificity: 59.4%), respectively. As shown in Figure 2, the AUROC for discrimination of surgical candidates (KL3 and KL4) was 0.844, 0.804, 0.766, and 0.718 for the combination of a single image with essential factors, single image with full factors, pairing images with essential factors, and pairing image with full factors, respectively. Pairwise DeLong test was applied to check the significance between models. The AUROC of single image + essential factors was 0.844, and significantly higher than pairing images + essential factors (ΔAUC = 0.078, z = 2.918, *p* = 0.004, 95% CI for the difference [0.026, 0.130]) and pairing images + full factors (ΔAUC = 0.126, z = 4.941, *p* < 0.001, 95% CI [0.076, 0.176]). Its difference from single image + full factors model was not statistically significant (ΔAUC = 0.040, z = 1.412, *p* = 0.158, 95% CI [−0.015, 0.094]). All models performed well above chance (AUC > 0.5).

**Figure 3:**
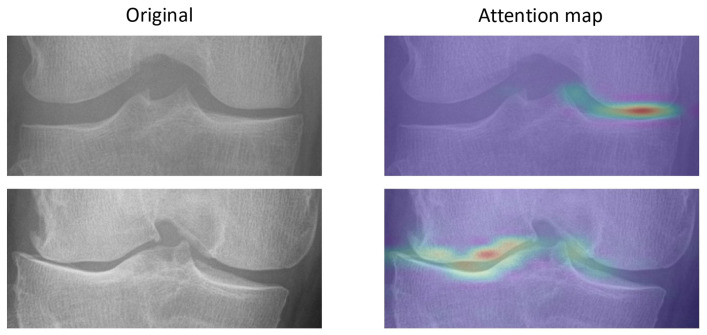
Attention map. The attention map shows that the model can identify OA features, including narrowing of joint space and emerging osteophytes, which are established radiographic markers of progression.

The attention maps show that OA features can be identified by the model, as shown in Figure 3. The attention maps demonstrate that the model demonstrates joint-space narrowing and emerging osteophytes, aligning with radiographic hallmarks of OA progression recognized by clinicians.

In external validation, as pairing images and full factors were unavailable, the combination of “single image with essential factors” was applied for further evaluation.

### 3.1. Model Performance in OAI Testing Set

The AUROC for discriminating OA progression was 0.808 in the OAI testing set. The overall accuracy of prediction in the OAI testing set was 74.1% with sensitivity 91.8% and specificity 71.0%. The odds ratio of positive prediction was 23.87 (CI: 11.24~50.67), as shown in Table 3.

### 3.2. Model Performance in External Validation

The AUROC for discriminating OA was 0.709 in the external validation set. The overall accuracy of prediction in the external validation set was 71.2% with sensitivity 72.2% and specificity 70.3%. The odds ratio of positive prediction was 5.92 (CI: 3.50~10.03), as shown in Table 3.

### 3.3. Odds Ratio of Traditional Factors and Model Prediction

The odds ratio of old age (≥70 years old) was 0.82 (CI: 0.46~1.44) in OAI and 1.44 (CI: 0.88~2.34) in the external validation set. The female sex had an odds ratio of 1.32 (CI: 0.82~2.12) in OAI and 0.89 (CI: 0.50~1.61) in external validation. The odds ratio of obesity (BMI ≥ 30) was 2.77 (CI: 1.17~6.55) and 1.29 (CI: 0.66~2.50) in OAI and external validation sets, respectively. In addition, as shown in Table 3, positive model prediction had an odds ratio of 23.87 (CI: 11.24~50.67) and 5.92 (CI: 3.50~10.03) in OAI and external validation sets, respectively.

## 4. Discussion

To identify the cases with a tendency to progress in the future is very useful in clinical scenarios. Although surgery is still the mainstay for severe knee OA, newer treatment strategies developed broadly in recent years. For example, conservative treatment such as CM-C have reported benefits in selected populations [15]. Accordingly, the proposed model is positioned to complement—not replace—clinician judgment by flagging individuals more likely to progress, thereby supporting individualized use of conservative options and identifying those who may ultimately require surgery.

Although big data carrying massive information content are the cornerstone of deep learning, complicated factors and expensive image studies for data input may be the barriers for developing an optimal model for clinical scenarios. Our study aimed to develop a practical OA prediction model using basic images and clinical data.

The ViT was applied for model training in the present study. The image features and impact of clinical factors were considered equally. To the best of our knowledge, the successful application of end-to-end radiographic-based OA prediction model in external validation has not been reported in previous studies.

Many risk factors of knee OA have been identified by previous studies, such as age, BMI, trauma, and previous knee surgery [4,5]. Zhang et al. created logistic regression models to predict knee OA progression using clinical factors. The AUROC is 0.60 for the OAI database. The models only included clinical parameters for prediction, and no image features were considered [16]. Complete factors of knee OA were included in our model as the input of “full factors”.

In addition to traditional risk factors, the genome for knee OA progression was reported in recent research. Kulm et al. presented a genome-wide association study on patients undergoing total knee arthroplasty (TKA). The study concluded that age and BMI have a more significant impact on the risk of end-stage OA than genetic factors. The genetic contribution to the development of severe disease is greater in younger patients [6]. In addition, several studies used MRI to predict OA progression based on the detailed information of degenerative meniscus and ligaments [9,10]. However, it is impractical to request a gene study or MRI report from each OA patient for a regular appointment in Taiwan.

Guan et al. have reported that CNN models have greater diagnostic performance compared with machine learning model and logistic regression using the OAI database [17]. They used Densenet as the backbone of the prediction model. The AUROC of predicting progression of joint space narrowing was 0.857. Leung et al. used OAI for model training to predict the probability of total knee arthroplasty in knee OA patients. The model performance was evaluated using AUROC, and the final result was 0.87 [18].

Prior deep-learning approaches for OA prognosis have largely relied on CNN-style image models or on generative forecasting of future radiographs. A recent diffusion-based method attains AUC ≈ 0.71 on OAI and improves interpretability by generating future images and knee landmarks, but it remains image-only and computationally heavier due to the generative stage [11]. In parallel, multimodal/tabular models using demographics, BMD, BMI, and radiographic grades (e.g., LGBM) achieve strong discrimination for specific structural phenotypes, yet they do not learn directly from raw images and require curated radiographic inputs [12]. Our work advances these lines by employing a Vision-Transformer that captures global joint context while fusing raw radiographs with clinical risk factors in a single end-to-end model. This design preserves interpretability via attention maps aligned with radiographic hallmarks and demonstrates cross-cohort generalization, addressing practical deployment beyond OAI.

Early intervention, including weight loss and quadriceps training, may slow OA progression. Thus, identification of the cases with the tendency to progress in the future is key to our model. Therefore, care should be taken to improve model performance for the group displaying progression tendency. We compared the combination between “single/pairing images” and “essential/full factors.” The result is displayed as a single image with essential factors providing better results in the progression group, as shown in Figure 2. Our study shows that massive input data can provide more information but not necessarily better results in the deep learning model.

We demonstrated the uncertainty of clinical factors and reliability in OA prediction. Traditionally, physicians use risk factors to evaluate knee OA progression by personal experience. The results of odds ratio for old age and female sex were not consistent in different databases. Although obesity showed higher risk of OA progression in both groups, only OAI showed statistical significance. The positive prediction in our model outperformed the conventional factors in both groups, as shown in Table 3. The result showed that image features extracted by the ViT model can provide more information about OA progression in addition to conventional risk factors.

As obesity is one of the most notable factors contributing to OA progression, different BMI distributions between testing sets were further evaluated in this study. We designed an optional step to adjust the diversity of the population. The results of ViT model and concatenation were adjusted by mean BMI, serving as the cutoff value, to balance the model performance in each group, as shown in Figure 1. The combination of single image and essential factors made the results between the progression and non-progression groups more balanced. The female sex and old age were also recognized as risk factors of knee OA [4]. However, the results of the odds ratio for gender and age were inconsistent between the testing sets. Age data and gender were applied in the pooling layer and then advanced to the MLP head. However, the model performance did not improve after adjusting for age and gender in the optional step.

We attribute the lower AUROC in external validation to dataset shift between cohorts—namely (1) differences in demographics and BMI distributions, (2) limited availability of paired radiographs and full clinical variables at the external site, (3) racial/ethnic composition differences, and (4) cohort-specific associations between risk factors and outcomes. These forms of covariate and prior-probability shift are well known to hinder cross-population generalization of deep models.

This study had several limitations. First, the follow-up duration used in model training was 48 months. Thus, cases with much longer or shorter follow-up duration may not be predicted precisely. Second, most OAI cases were Caucasian and African Americans, but almost all the cases in the external dataset were Asian (Taiwanese). The differences in race for OA progression have not been thoroughly studied before. Third, the necessity of different BMI cutoff values revealed that the model cannot be universally transferred to every population without risk adjustment. However, the flexible design of our algorithm can accommodate different populations and data, by means of adding pairing baseline images or risk adjustment after concatenation.

In summary, our ViT-based model provides the advantages of simplicity and flexibility and reliable prediction for knee OA cases. The model performance was excellent, especially in progression cases, potentially making early intervention in OA patients more efficient. Positive model prediction was proved with stronger power in assessing OA progression risk than traditional clinical factors. A prodigious database may be required for future studies in order to develop a universal OA prediction model without the necessity of adjustment.

## Figures and Tables

**Table 1:** Description of datasets used in this study.

Group	Database	Images	KL1	KL2	KL3
Train	OAI	4425	2450	1273	702
Validation	OAI	562	294	179	89
Test	OAI	578	332	179	67
External validation	Clinical images *	274	82	147	45

* From Far Eastern Memorial Hospital, New Taipei City, Taiwan. KL, Kellgren and Lawrence; Train, training data; OAI, Osteoarthritis Initiative.

**Table 2:** Comparison between OAI testing set and external validation.

	OAI Testing Set	External Validation
Images	578	274
Age (yr), mean ± SD	60.6 ± 9.3	71.4 ± 8.3
BMI (kg/m^2^), mean ± SD	28.6 ± 4.8	25.9 ± 3.9
Female gender (%)	58.9	79.2

SD: standard deviation.

**Table 3:** Model performance.

***Performance Comparison Between OAI and External Validation***		
	OAI testing set	External validation
Accuracy	74.1%	71.2%
Sensitivity	91.8%	72.2%
Specificity	71.0%	70.3%
AUROC (95%CI)	0.808	0.709
***Odds Ratio of Traditional Factors and Model Prediction***		
Elderly age	0.82 (CI: 0.46~1.44)	1.44 (CI: 0.88~2.34)
Female gender	1.32 (CI: 0.82~2.12)	0.89 (CI: 0.50~1.61)
Obesity	2.77 (CI: 1.17~6.55)	1.29 (CI: 0.66~2.50)
Positive model prediction	23.87 (CI: 11.24~50.67)	5.92 (CI: 3.50~10.03)

CI: 95% confidence intervals.

## Data Availability

Raw data are available in the osteoarthritis initiate (OAI) open dataset. Derived data supporting the findings of this study are available from the corresponding author on request due to ethical and privacy issue.
